# Evaluating a Deep Learning Diabetic Retinopathy Grading System Developed on Mydriatic Retinal Images When Applied to Non-Mydriatic Community Screening

**DOI:** 10.3390/jcm11030614

**Published:** 2022-01-26

**Authors:** Joan M. Nunez do Rio, Paul Nderitu, Christos Bergeles, Sobha Sivaprasad, Gavin S. W. Tan, Rajiv Raman

**Affiliations:** 1 Institute of Ophthalmology, University College London, London EC1V 9EL, UK; paulnderitu@nhs.net (P.N.); sobha.sivaprasad@nhs.net (S.S.); 2 Section of Ophthalmology, King’s College London, London WC2R 2LS, UK; 3 School of Biomedical Engineering & Imaging Sciences, King’s College London, London SE1 7EU, UK; christos.bergeles@kcl.ac.uk; 4 NIHR Moorfields Biomedical Research Centre, Moorfields Eye Hospital, London EC1V 2PD, UK; 5 Singapore Eye Research Institute, Singapore National Eye Center, Singapore 169856, Singapore; gavin.tan@singhealth.com.sg; 6 Duke-NUS Medical School, National University of Singapore, Singapore 169857, Singapore; 7 Sankara Nethralaya, 18, College Road, Chennai 600006, India; rajivpgraman@gmail.com

**Keywords:** diabetic retinopathy, handheld non-mydriatic images, deep learning

## Abstract

Artificial Intelligence has showcased clear capabilities to automatically grade diabetic retinopathy (DR) on mydriatic retinal images captured by clinical experts on fixed table-top retinal cameras within hospital settings. However, in many low- and middle-income countries, screening for DR revolves around minimally trained field workers using handheld non-mydriatic cameras in community settings. This prospective study evaluated the diagnostic accuracy of a deep learning algorithm developed using mydriatic retinal images by the Singapore Eye Research Institute, commercially available as Zeiss VISUHEALTH-AI DR, on images captured by field workers on a Zeiss Visuscout^®^ 100 non-mydriatic handheld camera from people with diabetes in a house-to-house cross-sectional study across 20 regions in India. A total of 20,489 patient eyes from 11,199 patients were used to evaluate algorithm performance in identifying referable DR, non-referable DR, and gradability. For each category, the algorithm achieved precision values of 29.60 (95% CI 27.40, 31.88), 92.56 (92.13, 92.97), and 58.58 (56.97, 60.19), recall values of 62.69 (59.17, 66.12), 85.65 (85.11, 86.18), and 65.06 (63.40, 66.69), and F-score values of 40.22 (38.25, 42.21), 88.97 (88.62, 89.31), and 61.65 (60.50, 62.80), respectively. Model performance reached 91.22 (90.79, 91.64) sensitivity and 65.06 (63.40, 66.69) specificity at detecting gradability and 72.08 (70.68, 73.46) sensitivity and 85.65 (85.11, 86.18) specificity for the detection of all referable eyes. Algorithm accuracy is dependent on the quality of acquired retinal images, and this is a major limiting step for its global implementation in community non-mydriatic DR screening using handheld cameras. This study highlights the need to develop and train deep learning-based screening tools in such conditions before implementation.

## 1. Introduction

There are 463 million people with diabetes in the world. 80% of this population reside in low- and middle-income countries (LMIC), where resources are limited, and 30% present diabetic retinopathy (DR) [1,2]. Regular screening for DR is recommended to identify vision-threatening DR (VTDR), an avoidable cause of blindness, and treat it promptly [3].

Many high-income countries have established DR screening as a public health programme, recommending yearly screening of people with diabetes [4]. DR screening is conducted at fixed locations, and images of the central retina acquired after pupil dilation by trained screeners using standardised, table-top fixed retinal cameras are graded for DR by qualified graders. Patients with VTDR, or if images are ungradable, are referred to ophthalmic departments for further management. This successful DR screening is laborious and cannot be translated to LMIC [5], where opportunistic DR screening is performed by minimally trained field workers in medical camps or public spaces, and pupil dilation is not routinely carried out due to restrictive policies. A major challenge of this strategy is the use of handheld retinal cameras without stabilising platforms through non-mydriatic pupils, which has been reported to drop the proportion of gradable images by about 20% [6]. Moreover, image acquisition by field workers in communities with limited healthcare access can be challenging due to the increased prevalence of undiagnosed co-pathologies, especially cataract [7].

One solution to improve the efficiency of DR screening programmes is to use automated algorithms to grade the retinal images. The successful advent of deep neural network (DNN) approaches in recent years has showcased a wide range of automated systems unfolding their benefits in several healthcare disciplines [8], and DR screening is no exception [9,10,11]. However, DNNs are trained on retinal images captured through dilated pupils to ensure high diagnostic accuracy. The algorithms are developed to identify referable images based on standard DR severity scales [12,13,14,15,16,17], such as the International Clinical Diabetic Retinopathy (ICDR) severity scale [18,19]. Some of these automated algorithms are already approved by regulators and implemented in a few screening programmes. Based on these reports, many manufacturers have also incorporated these algorithms into their low-cost cameras for instant offline grading of retinal images obtained from population-based screening in LMIC [17].

However, there is a paucity of studies that have evaluated the diagnostic accuracy of automated algorithms for the grading of retinal images captured on handheld cameras through non-mydriatic pupils [20,21]. Moreover, there are no reports of real-world implementation of automated grading in a multicentre DR screening programme in India.

In this study, we evaluated the diagnostic accuracy of the Deep Learning algorithm developed by the Singapore Eye Research Institute (SERI) on mydriatic retinal images, which is commercially deployed in Zeiss VISUHEALTH-AI DR, for grading non-mydriatic retinal images captured by field workers using handheld cameras through non-dilated pupils versus human graders in a real-world community DR screening in India. We report the performance of the automated DR system at predicting three possible outcomes: (1) referable DR, (2) non-referable DR, and (3) ungradable image. In addition, we evaluated the performance of the algorithm in detecting gradability (referable and non-referable) and eyes that require hospital referral defined as a total of ungradable and referable DR images. Finally, we report the regional variations in outcomes, intergrader agreement, and agreement between human graders and the algorithm.

## 2. Materials and Methods

### 2.1. Study Settings

Anonymised retinal images used in this study were captured as part of the SMART India study, a study that aimed to increase research capacity and capability to tackle the burden of blindness due to DR in India [22]. In this cross-sectional prospective and community-based study, door-to-door surveys and point of care non-laboratory tests, and retinal images were obtained using a non-mydriatic handheld fundus camera on people with diabetes in each household at 20 pre-defined sites (Table S1). Each site included both rural and urban areas across India. Field workers were trained to capture a set of at least two gradable retinal photographs from each eye through non-dilated eyes using the Zeiss Visuscout^®^ 100 camera. For each patient, a variable number of macula and optic disc images of each eye were taken to acquire the best possible images. When the acquisition of retinal images was not possible, potentially due to cataract or a small pupil, the same camera was used to take photographs of the anterior segment. Retinal fundus photographs were obtained from subjects who are known diabetics or who on the day of survey had a high random blood sugar of 160 mg/dL (8.9 mmol/L) or higher.

### 2.2. Image Grading by Graders (Reference Standard)

Retinal photographs captured by field workers were uploaded to a database for independent grading by primary (on-site) and secondary graders (in a Reading Centre). Each eye was independently graded by each grader, either a trained optometrist or ophthalmologist, and all images per eye were available to the graders. Senior ophthalmologists at each Reading Centre arbitrated discrepancies. Patient eyes were graded as no, mild, moderate, severe, and proliferative DR as per the ICDR severity scale [18,19], or as ungradable. The disease severity graded by the human graders for each patient eye was populated with three outcomes: (1) referable DR: moderate non-proliferative DR or worse with or without macular oedema (hard exudates/thickening around fovea), (2) non-referable DR: eyes with no DR or mild DR, and (3) ungradable. The final grade from human graders on the basis of all images per patient eye was used as the reference standard. Graders were masked with respect to the automated algorithm grades.

### 2.3. Imaging Grading by Automated Algorithm (Index Test)

VISUHEALTH-AI DR (Software version 1.8) is an automated screening web-service with an optimization algorithm using deep neural networks to automatically review patient’s fundus images for the presence of DR. The screening solution is indicated to categorize single-field macula-centred 40-degree non-mydriatic fundus images taken with VISUSCOUT 100 and delivers three possible outcomes: referable DR, non-referable DR, and ungradable image (with advice for hospital referral). VISUHEALTH-AI DR is a standalone health software product classified as a class IIa device as per Rule 11 of Medical Device Regulation (EU-MDR) 2017/745 Annex VIII.

Only colour retinal images that were macula-centred with a visible optic nerve head were selected from the pool of captured images to ensure that the performance of the algorithm was evaluated in accordance with the protocols used for its development. Optic disc-centred images and anterior segment photographs were discarded (Figure S1). The algorithm grading was independent of the human grading.

### 2.4. Outcomes

The fully anonymised/deidentified images available for each patient eye were independently analysed. Image-level outputs were processed and tabulated for the three possible outcomes to obtain the eye-level prediction for evaluation against the reference standard. Patient-eye predictions were derived as follows:

At least one referable image derived in a referable patient eye;
Non-referable and ungradable (if any) images derived in a non-referable patient eye;
Two or more ungradable images derived in an ungradable patient eye.

Performance was evaluated as a three outcome multilabel system as well as at two other relevant binary tasks: “gradability” and “hospital-referable”. Gradability assessment evaluated device performance at discerning gradable images (referable + non-referable) from ungradable images. Hospital-referable assessment evaluated the model’s ability to discern samples that must be sent for further screening, i.e., referable + ungradable from non-referable. Site, age category, and visual acuity covariates were also studied.

### 2.5. Statistical Analysis

A descriptive analysis of the participant demographics by site (20 sites), age categories (≤40, 41–60, 61–70, and >70 years), and visual acuity (VA) categories (Normal: logMAR VA < 0.4, moderate visual impairment (VI): logMAR 0.4 ≤ VA < 1.0, severe VI: logMAR 1.0 ≤ VA < 1.3, blind: logMAR VA ≥ 1.3 [23]) is performed. The robustness of the algorithm at the different tasks was evaluated by comparing the standard reference to the automated prediction. For the main multiclass task, a full metric report was calculated with precision (positive predictive value), recall, and F-score. For the binary tasks of gradability and hospital-referable performance, sensitivity and specificity metrics were calculated. All reports were also studied for each site, age category, and visual acuity categories. Interobserver variability was measured with the quadratic weighted Kappa scores (see Table S2 for metric definitions). In all cases, exact Clopper–Pearson 95% confidence intervals were calculated.

## 3. Results

From a pool of 60,633 retinal fundus images, a total of 29,656 images from 11,199 patients and 20,489 patient eyes were eligible for the study (Figure S1). Table 1 shows participant demographics and eye grade distribution at each site. The average age of the participants was 57.7 (11.1) years, with 5365 males (47.9%). Sites 18 and 14 had the highest and lowest average age of 66.7 (12.3) and 53.7 (8.1) years, respectively. The participants were also categorised by age and visual acuity categories (Table S3).

The overall and per-site algorithm performance metrics are listed in Table 2. Precision values reached 29.6 (95% CI 27.40, 31.88), 92.56 (92.13, 92.97), and 58.58 (56.97, 60.19), with recall values of 62.69 (59.17, 66.12), 85.65 (85.11, 86.18), and 65.06 (63.40, 66.69), and F-scores of 40.22 (38.25, 42.21), 88.97 (88.62, 89.31), and 61.65 (60.50, 62.80), for referable, non-referable, and ungradable categories, respectively. Site 7 showed the highest F-score for referable category, reaching 66.3 (59.25, 72.90). Among the age categories (Table S4), the highest F-score was 47.39 (44.60, 50.18) for 41–60 years. Regarding visual acuity (Table S4), the highest F-score for referable cases, for severe VI patients, was 66.67 (49.03, 81.44).

Overall performance at the gradability task (Table 3) was 91.22 (95% CI 90.79, 91.64) sensitivity and 65.06 (63.40, 66.69) specificity. The best performance was shown by site 2, with 89.45 (87.85, 90.90) sensitivity and 92.31 (87.81, 95.54) specificity, and site 6, with 88.60 (86.38, 90.57) sensitivity and 94.68 (88.02, 98.25) specificity. Among age categories, 41–60 years showed the highest sensitivity of 93.13 (92.64, 93.59), with 61.36 (58.51, 64.15) specificity. For visual acuity, the Normal category reported the highest sensitivity of 91.41 (90.93, 91.87), with a specificity of 62.15 (60.06, 64.21).

At the hospital-referable task (Table 3), overall performance reached 72.08 (95% CI 70.68, 73.46) sensitivity and 85.65 (85.11, 86.18) specificity. Site 11, with 91.40 (83.75, 96.21) sensitivity and 87.64 (83.15, 91.28) sensitivity, and site 19, with 94.74 (82.25, 99.36) sensitivity and 88.31 (85.68, 90.61) sensitivity, showed noticeably higher performance. Age category of >70 showed the highest sensitivity of 75.44 (72.68, 78.06), with 74.61 (72.45, 76.68) specificity. Severe VI category reported the highest sensitivity of 87.06 (78.02, 93.36), with 71.57 (61.78, 80.06) of specificity.

### Grader and Model Assessment

As listed in Table 4, the algorithm reported a Kappa value of 0.47 (95% CI 0.44, 0.50) for referable DR. For the same task, primary and secondary graders showed an agreement of 0.60 (0.57, 0.63) Kappa. When final grades (reference standard, after arbitration) were compared to primary and secondary graders, Kappa values were 0.66 (0.64, 0.69) and 0.84 (0.83, 0.86), respectively.

At the gradability task, model performance reached a Kappa value of 0.54 (95% CI 0.52, 0.56), and agreement between primary and secondary graders reached 0.60 (0.58, 0.61). Primary and secondary comparison to the reference standard showed 0.67 (0.65, 0.68) and 0.84 (0.83, 0.85), respectively. At the hospital-referable task, the model reached a Kappa value of 0.51 (0.50, 0.53), whereas primary and secondary comparison reached 0.60 (0.58, 0.61). Primary and secondary graders reached 0.67 (0.65, 0.68) and 0.84 (0.83, 0.85), respectively, when compared to the final reference standard.

## 4. Discussion

**Figure 1:**
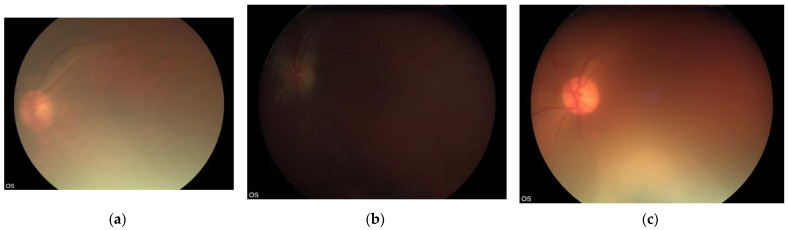
Image examples of disagreement. (**a**) Primary grader: ungradable, secondary grader: ungradable, final grade: ungradable, VISUHEALTH-AI DR: referable DR. (**b**) Primary grader: non-referable DR, secondary grader: ungradable, final grade: non-referable DR, VISUHEALTH-AI DR: ungradable. (**c**) Primary grader: non-referable DR, secondary grader: ungradable, final grade: ungradable, VISUHEALTH-AI DR: non-referable DR.

We evaluated the accuracy of an offline automated screening algorithm to identify referable DR from fundus images of people with diabetes captured by minimally trained field workers using non-mydriatic handheld cameras in a home environment. To our knowledge, this is the first prospective multicentre study on a considerably large dataset of handheld retinal images taken by multiple field workers in a community setting that mirrors the real-life implementation of such programmes in LMIC. We show that the success of an automated AI algorithm is dependent on the quality of the acquired retinal images. Although validation studies to date have shown that most automatic algorithms where mydriatic fundus images were used have high diagnostic accuracy, our study shows that real-world scenarios of DR screening in India where non-mydriatic DR screening is widely practised pose challenges (Figure 1).

Most previous algorithms for detecting referable DR have used mydriatic retinal photographs taken as part of in-clinic screening programmes and acquired using table-top retinal cameras, which contributes to significant dataset differences in terms of image quality. Gulshan et al. reported, for referable DR, a 90.3% sensitivity and 98.1% specificity for the EyePACS-1 dataset. Similarly, Gargeya et al. [14] showed a sensitivity of 93% and a specificity of 87% on the Messidor-2 dataset [24], and a study by Ting et al. [13] reported a sensitivity of 90.5% and a specificity of 91.6% on their primary validation dataset. More recently, Gulshan et al. [17] presented a prospective study on in-clinic non-mydriatic images from two different sites. The automated DR detection was equal to or worse than the manual grading, with 88.9% sensitivity and 92.2% specificity at one site and 92.1% sensitivity and 95.2% specificity at the other sites, highlighting the difference in performance between in-clinic and community performance. Few studies have evaluated automated systems for referable DR detection in handheld retinal images or in community settings. Rajalakshmi et al. [20] presented a study on 2408 smartphone-based mydriatic fundus photographs taken by hospital trained staff in a clinic environment and reported sensitivity of 95.8% and specificity 80.2% at detecting any DR. Similarly, Natarajan et al. presented a pilot study on 223 patients where a smartphone-based automated system was used to detect referable DR. The authors reported 100.0% sensitivity and 88.4% specificity [21].

It is important to highlight that our investigation was substantially different from previous studies, and, therefore, comparisons are not straightforward. Grading non-mydriatic retinal images captured by field workers using a handheld camera in the patient’s home entails application-specific challenges.

Mydriatic retinal photography, where resources are available, has been widely shown as the most effective screening strategy to provide high-quality DR screening since it increases image quality and allows for higher sensitivities [25,26]. On the contrary, non-mydriatic retinal imaging increases failure screening rates resulting from media opacity or small pupils [1]. However, DR screening without mydriasis in primary care premises have been proven to be a valid cost-effective screening method with advantages, not only for patient convenience, but also for logistic reasons [27]. These advantages facilitate the development of community-based screening programs in LMIC. Nevertheless, our study results show that by taking image acquisition out of controlled stress-free hospital premises, new challenges are generated.

In our study, the evaluation of the specific tasks of gradable and hospital-referable eye detection showed higher performance, suggesting that there is more consensus between human graders and the algorithm in identifying gradable images. In particular, hospital-referable is showed as the most separable class. This is an encouraging outcome, given that both referable DR and ungradable patients must be sent to ophthalmic departments for further management, which makes hospital-referable patient detection the most crucial task when using these automatic algorithms in such DR screening programmes. Although this process will ensure safety, increased referral to hospitals will overburden the already stretched ophthalmic services. In addition, we are reliant on patients attending for retinal examination after pupil dilation, and so, the overall effectiveness of such DR screening programmes may be compromised.

Comparatively, the study shows that identifying referable DR is the most challenging task. Our study highlights the limitations of the algorithm to diagnose referable DR in these community screening settings when handheld cameras are used and the pupils are not dilated. This is of significance for policy-makers in LMIC due to restrictions placed on dilating pupils in many of these countries. It should be noted that human graders had access to multiple fields for grading, whereas the algorithm, following its specifications, was only presented with single field fundus images, and this can be known to reduce sensitivity and specificity, particularly in non-mydriatic photography [28,29,30].

Algorithm evaluation by site also showed remarkable variations in performance. Different acquisition settings cause varying quality photographs, which can have a significant impact in referable DR screening and automated prediction algorithms. This is also reflected by the percentage of ungradable images, reaching up to over 30% in some sites.

We also investigated the performance of the algorithm by age and visual acuity of the screened individuals. Varying degrees of performance are shown for the different outcomes analysed. Performance differences for age and visual acuity categories are less remarkable in gradability and hospital-referable tasks. The detection of referable DR was less accurate in older individuals, which may be related to the poorer image quality caused by higher prevalence of co-pathology, especially cataract and small pupils. However, performance by visual acuity did not show the same trend, suggesting that variable image quality may be more related to technical challenges faced by the field workers.

We compared automated grading performance with the intergrader agreement among human graders. Agreement between human graders was also only moderate for all outcomes, which is concordant with the variable agreement reported by previous studies [31,32]. A higher agreement was observed between arbitration graders (who had access to all grades) and secondary graders than primary graders. Automated grading and human grading showed the lowest agreement, with the gradability task reporting the highest Kappa values.

The main limitation of the study is the unbalanced grading setting between human graders and the algorithm. VISUHEALTH-AI DR is designed to categorize single field macula-centred fundus photographs. However, field workers captured a set of two or more retinal photographs that, in all cases, included at least a macula-centred and an optic nerve head centred image. As a result, the algorithm’s prediction is hindered by a partial use of the data that was available to human graders. These study settings allow us to establish the results as the worst-case scenario for the evaluation of algorithm performance and unveils margins for improvement to be explored.

In the future, supervised machine learning methods for DR evaluation must demonstrate robustness of retinal image datasets acquired in various settings of DR screening to enable widespread implementation and to reduce health inequality. Investigation of automated referable DR systems in community settings with non-mydriatic retinal imaging is a key requirement to develop resource-driven screening programs in LMICs. Although we strongly recommend mydriatic retinal photography captured on fixed cameras, it is not logistically possible to ensure global coverage of DR screening with such methodologies. Several strategies are required to ensure regular DR screening of people with diabetes around the world, especially when one-tenth of the global population is estimated to have diabetes by 2040, with 80% of them living in countries with limited resources. Manufacturers should follow a two-step strategy when they incorporate automated algorithms in retinal cameras. The first is to automatically qualify the gradability of a retinal image for its eligibility for automated grading and then to apply the automated algorithm only if a retinal image passes the gradability test [33]. To ensure that automated grading can be implemented globally, images from real-life programmes from LMIC reflecting their specific acquisition conditions should be used in the development of automated algorithms to allow models to learn their distinct features and leverage that crucial knowledge.

In conclusion, although AI may be more efficient in grading large numbers of retinal images, the quality of captured images in real-world community settings determines the success of any AI system used for non-mydriatic DR screening. To be implemented globally, AI systems should leverage that specific knowledge and use images acquired in such conditions in their development process. In this study, we analysed the performance of the Zeiss VISUHEALTH-AI DR algorithm, developed by SERI, under such premises.

## Figures and Tables

**Table 1:** Patient demographics for each site and patient eye grade distribution.

Sites	Images, Total No. (%) ^a^	Images per Patient Eye, Mean (SD) ^b^	Patients, Total No. (%) ^a^	Age, Mean (SD) ^b^	Male, Total No. (%) ^b^	Female, Total No. (%) ^b^	Patient Eyes, Total No. (%) ^a^	Referable, Total No. (%) ^b^	Non-Referable, Total No. (%) ^b^	Ungradable, Total No. (%) ^b^
**1**	2408 (8.1)	1.5 (0.7)	836 (7.5)	54.2 (10.3)	402 (48.1)	434 (51.9)	**1576 (7.7)**	23 (1.5)	1404 (89.1)	149 (9.5)
**2**	2268 (7.6)	1.2 (0.5)	994 (8.9)	54.1 (10.0)	537 (54.0)	456 (45.9)	**1829 (8.9)**	108 (5.9)	1513 (82.7)	208 (11.4)
**3**	730 (2.5)	1.3 (0.5)	436 (3.9)	55.4 (10.7)	166 (38.1)	270 (61.9)	**576 (2.8)**	12 (2.1)	354 (61.5)	210 (36.5)
**4**	1308 (4.4)	1.3 (0.5)	549 (4.9)	55.5 (11.1)	304 (55.4)	245 (44.6)	**1036 (5.1)**	14 (1.4)	906 (87.5)	116 (11.2)
**5**	338 (1.1)	1.4 (0.6)	170 (1.5)	54.2 (10.8)	109 (64.1)	59 (34.7)	**231 (1.1)**	4 (1.7)	193 (83.5)	34 (14.7)
**6**	1315 (4.4)	1.3 (0.5)	557 (5.0)	55.1 (10.1)	315 (56.6)	242 (43.4)	**1024 (5.0)**	50 (4.9)	880 (85.9)	94 (9.2)
**7**	1225 (4.1)	1.1 (0.4)	569 (5.1)	56.4 (11.5)	355 (62.4)	214 (37.6)	**1075 (5.2)**	91 (8.5)	845 (78.6)	139 (12.9)
**8**	2110 (7.1)	1.2 (0.4)	943 (8.4)	61.9 (9.8)	369 (39.1)	571 (60.6)	**1812 (8.8)**	125 (6.9)	1475 (81.4)	212 (11.7)
**9**	2952 (10.0)	2.1 (0.9)	752 (6.7)	57.4 (10.1)	260 (34.6)	492 (65.4)	**1436 (7.0)**	22 (1.5)	1096 (76.3)	318 (22.1)
**10**	3635 (12.3)	1.9 (0.4)	1066 (9.5)	62.9 (10.5)	416 (39.0)	650 (61.0)	**1946 (9.5)**	49 (2.5)	1141 (58.6)	756 (38.8)
**11**	714 (2.4)	1.9 (1.0)	212 (1.9)	56.7 (9.5)	90 (42.5)	122 (57.5)	**368 (1.8)**	21 (5.7)	275 (74.7)	72 (19.6)
**12**	1668 (5.6)	1.2 (0.4)	757 (6.8)	58.4 (11.4)	389 (51.4)	367 (48.5)	**1427 (7.0)**	12 (0.8)	1145 (80.2)	270 (18.9)
**13**	751 (2.5)	1.3 (0.5)	318 (2.8)	54.4 (10.4)	133 (41.8)	185 (58.2)	**591 (2.9)**	20 (3.4)	523 (88.5)	48 (8.1)
**14**	703 (2.4)	1.2 (0.5)	327 (2.9)	53.7 (8.1)	232 (70.9)	94 (28.7)	**585 (2.9)**	22 (3.8)	549 (93.8)	14 (2.4)
**15**	662 (2.2)	1.4 (0.6)	286 (2.6)	55.8 (10.4)	170 (59.4)	116 (40.6)	**480 (2.3)**	10 (2.1)	384 (80.0)	86 (17.9)
**16**	647 (2.2)	1.1 (0.4)	336 (3.0)	56.3 (9.4)	184 (54.8)	151 (44.9)	**587 (2.9)**	17 (2.9)	557 (94.9)	13 (2.2)
**17**	1486 (5.0)	2.0 (0.5)	380 (3.4)	57.1 (11.2)	181 (47.6)	198 (52.1)	**725 (3.5)**	36 (5.0)	612 (84.4)	77 (10.6)
**18**	1092 (3.7)	1.0 (0.2)	586 (5.2)	66.7 (12.3)	92 (15.7)	494 (84.3)	**1044 (5.1)**	17 (1.6)	685 (65.6)	342 (32.8)
**19**	2149 (7.2)	2.9 (1.2)	377 (3.4)	54.5 (9.0)	235 (62.3)	142 (37.7)	**731 (3.6)**	27 (3.7)	693 (94.8)	11 (1.5)
**20**	1495 (5.0)	1.1 (0.2)	748 (6.7)	59.9 (11.2)	426 (57.0)	322 (43.0)	**1410 (6.9)**	92 (6.5)	1204 (85.4)	114 (8.1)
**Total**	29,656	1.4 (0.7)	11,199	57.7 (11.1)	5365 (47.9)	5824 (52.0)	**20,489**	772 (3.8)	16,434 (80.2)	3283 (16.0)

^a^ Percentages correspond to the final dataset (total). ^b^ Mean (SD) and percentages correspond to each site. Age and gender were available for 11,191 and 11,189 patients, respectively.

**Table 2:** Patient VISUHEALTH-AI DR performance evaluation by site.

	Precision			Recall			F-Score		
	Referable	Non-Referable	Ungradable	Referable	Non-Referable	Ungradable	Referable	Non-Referable	Ungradable
**1**	19.30 (10.05–31.91)	99.70 (99.13–99.94)	28.32 (24.48–32.41)	47.83 (26.82–69.41)	71.01 (68.56–73.37)	98.66 (95.24–99.84)	27.50 (18.10–38.62)	82.95 (81.38–84.43)	44.01 (40.21–47.87)
**2**	54.05 (44.33–63.55)	96.09 (94.91–97.06)	52.89 (47.61–58.12)	55.56 (45.68–65.12)	86.05 (84.21–87.76)	92.31 (87.81–95.54)	54.79 (47.95–61.51)	90.79 (89.68–91.83)	67.25 (63.23–71.09)
**3**	19.05 (5.45–41.91)	87.96 (83.50–91.56)	61.92 (55.96–67.62)	33.33 (9.92–65.11)	68.08 (62.95–72.91)	82.86 (77.07–87.7)	24.24 (11.09–42.26)	76.75 (73.25–80.00)	70.88 (66.64–74.86)
**4**	23.26 (11.76–38.63)	96.84 (95.47–97.89)	79.25 (70.28–86.51)	71.43 (41.90–91.61)	94.81 (93.16–96.16)	72.41 (63.34–80.3)	35.09 (22.91–48.87)	95.82 (94.78–96.70)	75.68 (69.48–81.17)
**5**	18.18 (2.28–51.78)	96.23 (91.97–98.60)	47.54 (34.60–60.73)	50.00 (6.76–93.24)	79.27 (72.87–84.76)	85.29 (68.94–95.05)	26.67 (7.79–55.10)	86.93 (82.96–90.27)	61.05 (50.50–70.89)
**6**	32.18 (22.56–43.06)	97.44 (96.03–98.45)	45.64 (38.51–52.91)	56.00 (41.25–70.01)	82.16 (79.47–84.63)	94.68 (88.02–98.25)	40.88 (32.56–49.60)	89.15 (87.53–90.62)	61.59 (55.72–67.23)
**7**	61.90 (51.91–71.21)	92.87 (90.94–94.51)	83.33 (75.20–89.66)	71.43 (61.00–80.41)	94.08 (92.27–95.58)	68.35 (59.92–75.97)	66.33 (59.25–72.90)	93.47 (92.19–94.60)	75.10 (69.30–80.30)
**8**	40.66 (33.45–48.17)	97.25 (96.14–98.11)	41.54 (37.03–46.16)	59.20 (50.05–67.90)	76.68 (74.43–78.82)	91.51 (86.91–94.89)	48.21 (42.50–53.95)	85.75 (84.35–87.06)	57.14 (53.32–60.90)
**9**	13.48 (8.31–20.24)	90.37 (88.45–92.06)	83.26 (77.58–87.99)	86.36 (65.09–97.09)	89.05 (87.05–90.84)	56.29 (50.64–61.82)	23.31 (17.06–30.57)	89.71 (88.35–90.95)	67.17 (63.00–71.14)
**10**	22.73 (16.37–30.16)	73.65 (71.25–75.95)	86.85 (83.15–89.99)	71.43 (56.74–83.42)	89.66 (87.74–91.36)	46.3 (42.70–49.93)	34.48 (27.97–41.46)	80.87 (79.28–82.39)	60.4 (57.51–63.23)
**11**	44.19 (29.08–60.12)	96.79 (93.77–98.60)	77.63 (66.62–86.40)	90.48 (69.62–98.83)	87.64 (83.15–91.28)	81.94 (71.11–90.02)	59.38 (46.37–71.49)	91.98 (89.32–94.16)	79.73 (72.34–85.89)
**12**	9.16 (4.82–15.45)	90.55 (88.74–92.16)	93.39 (87.39–97.10)	100.0 (73.54–100.0)	92.93 (91.28–94.34)	41.85 (35.90–47.98)	16.78 (11.06–23.94)	91.72 (90.53–92.81)	57.80 (52.73–62.75)
**13**	20.69 (11.17–33.35)	94.33 (91.91–96.20)	58.97 (42.10–74.43)	60.00 (36.05–80.88)	89.10 (86.11–91.64)	47.92 (33.29–62.81)	30.77 (20.81–42.24)	91.64 (89.77–93.27)	52.87 (41.87–63.67)
**14**	28.33 (17.45–41.44)	99.17 (97.89–99.77)	25.58 (13.52–41.17)	77.27 (54.63–92.18)	87.07 (83.97–89.76)	78.57 (49.20–95.34)	41.46 (30.68–52.88)	92.73 (90.97–94.24)	38.60 (26.00–52.43)
**15**	28.57 (13.22–48.67)	88.03 (84.44–91.04)	72.55 (58.26–84.11)	80.00 (44.39–97.48)	91.93 (88.74–94.45)	43.02 (32.39–54.15)	42.11 (26.31–59.18)	89.94 (87.62–91.95)	54.01 (45.30–62.56)
**16**	19.70 (10.93–31.32)	99.16 (97.87–99.77)	25.58 (13.52–41.17)	76.47 (50.10–93.19)	85.10 (81.87–87.95)	84.62 (54.55–98.08)	31.33 (21.59–42.44)	91.59 (89.73–93.21)	39.29 (26.50–53.25)
**17**	32.63 (23.36–43.02)	92.98 (90.62–94.89)	93.75 (79.19–99.23)	86.11 (70.5–95.33)	90.85 (88.28–93.01)	38.96 (28.05–50.75)	47.33 (38.55–56.23)	91.90 (90.22–93.38)	55.05 (45.22–64.59)
**18**	20.00 (11.65–30.83)	84.59 (81.73–87.16)	83.53 (78.40–87.86)	88.24 (63.56–98.54)	88.18 (85.52–90.5)	62.28 (56.91–67.44)	32.61 (23.20–43.18)	86.35 (84.44–88.10)	71.36 (67.55–74.95)
**19**	27.17 (18.42–37.45)	99.67 (98.83–99.96)	40.00 (21.13–61.33)	92.59 (75.71–99.09)	88.31 (85.68–90.61)	90.91 (58.72–99.77)	42.02 (33.03–51.41)	93.65 (92.19–94.91)	55.56 (38.10–72.06)
**20**	32.00 (21.69–43.78)	93.62 (92.01–95.00)	40.51 (34.20–47.05)	26.09 (17.48–36.29)	85.38 (83.26–87.33)	84.21 (76.2–90.37)	28.74 (22.01–36.24)	89.31 (87.98–90.55)	54.70 (49.33–59.99)
**Total**	29.6 (27.40–31.88)	92.56 (92.13–92.97)	58.58 (56.97–60.19)	62.69 (59.17–66.12)	85.65 (85.11–86.18)	65.06 (63.40–66.69)	40.22 (38.25–42.21)	88.97 (88.62–89.31)	61.65 (60.50–62.80)

**Table 3:** VISUHEALTH-AI DR gradability and hospital-referable performance evaluation by site, age category, and visual acuity.

		Gradability		Hospital-Referable	
		Sensitivity (95% CI)	Specificity (95% CI)	Sensitivity (95% CI)	Specificity (95% CI)
**Site**	**1**	73.93 (71.57–76.19)	98.66 (95.24–99.84)	98.26 (94.99–99.64)	71.01 (68.56–73.37)
	**2**	89.45 (87.85–90.9)	92.31 (87.81–95.54)	83.23 (78.64–87.18)	86.05 (84.21–87.76)
	**3**	70.77 (65.81–75.38)	82.86 (77.07–87.7)	85.14 (79.76–89.54)	68.08 (62.95–72.91)
	**4**	97.61 (96.4–98.5)	72.41 (63.34–80.3)	78.46 (70.4–85.19)	94.81 (93.16–96.16)
	**5**	83.76 (77.85–88.62)	85.29 (68.94–95.05)	84.21 (68.75–93.98)	79.27 (72.87–84.76)
	**6**	88.6 (86.38–90.57)	94.68 (88.02–98.25)	86.81 (80.16–91.87)	82.16 (79.47–84.63)
	**7**	97.97 (96.85–98.77)	68.35 (59.92–75.97)	73.48 (67.28–79.06)	94.08 (92.27–95.58)
	**8**	82.94 (81.0–84.75)	91.51 (86.91–94.89)	90.5 (86.86–93.41)	76.68 (74.43–78.82)
	**9**	96.78 (95.57–97.73)	56.29 (50.64–61.82)	69.41 (64.21–74.27)	89.05 (87.05–90.84)
	**10**	95.55 (94.21–96.65)	46.3 (42.7–49.93)	54.53 (51.02–58.01)	89.66 (87.74–91.36)
	**11**	94.26 (90.96–96.62)	81.94 (71.11–90.02)	91.4 (83.75–96.21)	87.64 (83.15–91.28)
	**12**	99.31 (98.64–99.7)	41.85 (35.9–47.98)	60.64 (54.67–66.38)	92.93 (91.28–94.34)
	**13**	97.05 (95.26–98.31)	47.92 (33.29–62.81)	58.82 (46.23–70.63)	89.10 (86.11–91.64)
	**14**	94.4 (92.18–96.14)	78.57 (49.2–95.34)	88.89 (73.94–96.89)	87.07 (83.97–89.76)
	**15**	96.45 (94.11–98.04)	43.02 (32.39–54.15)	50.00 (39.62–60.38)	91.93 (88.74–94.45)
	**16**	94.43 (92.22–96.16)	84.62 (54.55–98.08)	86.67 (69.28–96.24)	85.1 (81.87–87.95)
	**17**	99.69 (98.89–99.96)	38.96 (28.05–50.75)	62.83 (53.24–71.74)	90.85 (88.28–93.01)
	**18**	94.02 (92.0–95.65)	62.28 (56.91–67.44)	69.36 (64.31–74.09)	88.18 (85.52–90.5)
	**19**	97.92 (96.59–98.83)	90.91 (58.72–99.77)	94.74 (82.25–99.36)	88.31 (85.68–90.61)
	**20**	89.12 (87.3–90.76)	84.21 (76.2–90.37)	66.02 (59.11–72.46)	85.38 (83.26–87.33)
**Age**	**≤40**	91.61 (88.57–94.05)	66.67 (34.89–90.08)	63.64 (40.66–82.8)	91.41 (88.3–93.91)
	**41–60**	93.13 (92.64–93.59)	61.36 (58.51–64.15)	69.57 (67.28–71.79)	89.21 (88.6–89.79)
	**61–70**	88.14 (87.11–89.12)	65.41 (62.57–68.17)	72.72 (70.28–75.06)	80.09 (78.79–81.35)
	**>70**	86.35 (84.64–87.93)	69.30 (66.26–72.23)	75.44 (72.68–78.06)	74.61 (72.45–76.68)
**Visual Acuity**	**Normal**	91.41 (90.93–91.87)	62.15 (60.06–64.21)	69.39 (67.61–71.12)	86.73 (86.14–87.3)
	**VI**	90.71 (89.59–91.74)	67.95 (64.81–70.98)	75.27 (72.62–77.79)	81.56 (80.04–83.01)
	**Severe VI**	82.46 (74.21–88.94)	80.82 (69.92–89.1)	87.06 (78.02–93.36)	71.57 (61.78–80.06)
	**Blind**	78.79 (70.82–85.42)	81.43 (73.98–87.5)	86.84 (80.41–91.77)	65.00 (55.76–73.48)
**All data**		91.22 (90.79–91.64)	65.06 (63.40–66.69)	72.08 (70.68–73.46)	85.65 (85.11–86.18)

**Table 4:** Quadratic weighted Kappa scores.

	Quadratic Weighted K (95% CI)		
	Referable DR	Gradability	Hospital Referable
Primary vs. Secondary	0.60 (0.57, 0.63)	0.60 (0.58, 0.61)	0.60 (0.58, 0.61)
Final grade (GT) vs. Primary	0.66 (0.64, 0.69)	0.67 (0.65, 0.68)	0.67 (0.66, 0.69)
Final grade (GT) vs. Secondary	0.84 (0.83, 0.86)	0.84 (0.83, 0.85)	0.84 (0.84, 0.85)
VISUHEALTH-AI DR vs. GT	0.47 (0.44, 0.50)	0.54 (0.52, 0.56)	0.51 (0.50, 0.53)

## Data Availability

The curated anonymised dataset is available to researchers on application to the Moorfields Eye Hospital Research Management Committee for data access after sufficient regulatory approval is obtained.

## Supplementary Materials

The following are available online at https://www.mdpi.com/article/10.3390/jcm11030614/s1, Table S1: SMART-India study sites, Table S2: Performance metrics, Table S3: Patient eyes by age categories and visual acuity, Table S4: Performance evaluation by age category and visual acuity, Figure S1: Study participants and data curation.
