# Supplementary material for: Evaluating a Deep Learning Diabetic Retinopathy Grading System Developed on Mydriatic Retinal Images When Applied to Non-Mydriatic Community Screening
Source: J Clin Med. 2022 Jan 26;11(3):614. doi: 10.3390/jcm11030614 (PMC8836386; doi:10.3390/jcm11030614)
Supplement: Supplementary file 1 [file jcm-11-00614-s001.zip › jcm-1499832-supplementary.pdf]

Journal of
Clinical Medicine

MDPI

Supplementary Materials

Supplement to: Evaluating a deep learning diabetic retinopathy grading system developed on mydriatic retinal images when applied to non-mydriatic community screening. Nunez do Rio JM, Nderitu P, Bergeles C, Sivaprasad S, Tan GSW, Raman R.

Table S1. SMART-India study sites (Location, ID).

Chennai (1)	Bangalore (6)	New Delhi (11)	Raipur (16)
Chennai (2)	Coimbatore (7)	Bhubaneswar (12)	Haldia (17)
Madurai (3)	Cochin (8)	Guwahati (13)	Angamaly (18)
Hyderabad (4)	Mumbai (9)	Bhopal (14)	Jalna (19)
Kolkata (5)	Kerala (10)	Chitrakoot (15)	Pune (20)

Table S2. Performance metrics. Precision, recall and F-score were used to analyse the performance of the multiclass algorithm. Sensitivity and specificity (recall of the positive and the negative class, respectively) were used to evaluate the performance of the binary tasks. The positive classes in the two binary tasks analysed were considered to be gradable and hospital-referable. k is the quadratic weighted Cohen's Kappa (where n is the number of classes, x_{ij} is the matrix $n \times n$ of observed values, y_{ij} is the matrix $n \times n$ of expected values based on chance agreement, and $w_{ij} = (i - j/n - 1)^2$ for $0 \leq i, j < n$ is the matrix $n \times n$ of weights).

$Precision = \frac{TruePositives}{TruePositives + FalsePositives}$
$Recall = \frac{TruePositives}{TruePositives + FalseNegatives}$
$F - score = 2\frac{Precision \cdot Recall}{Precision + Recall}$
$Sensitivity = \frac{TruePositives}{TruePositives + FalseNegatives}$
$Specificity = \frac{TrueNegatives}{TrueNegatives + FalsePositives}$
$k = 1 - \frac{\sum_{i=1}^{n}\sum_{j=1}^{n} w_{ij}x_{ij}}{\sum_{i=1}^{n}\sum_{j=1}^{n} w_{ij}y_{ij}}$

Table S3. Patient eyes by age categories and visual acuity.

	**Age**				**Visual acuity[a]**			
	**≤40**	**41-60**	**61-70**	**>70**	**Normal**	**VI**	**Severe VI**	**Blind**
**Referable (%)**	10 (2.3)	463 (3.8)	229 (4.4)	70 (2.6)	547 (3.4)	200 (5.3)	12 (6.4)	12 (4.4)
**Non-referable (%)**	419 (95.0)	10,507 (86.5)	3,828 (73.6)	1,666 (62.1)	13,260 (83.1)	2,695 (70.9)	102 (54.5)	120 (44.1)
**Ungradable (%)**	12 (2.7)	1,180 (9.7)	1,142 (22.0)	948 (35.3)	2,148 (13.5)	908 (23.9)	73 (39.0)	140 (51.5)
**Patient eyes, total No**	441	12,150	5,199	2,684	15,955	3,803	187	272

[a] Normal: logMAR VA<0.4, Visual impairment (VI): logMAR 0.4≤VA<1.0, Severe VI: logMAR1.0≤VA<1.3, Blind: logMar VA≥1.3.

Table S4. VISUHEALTH – AI DR performance evaluation by age category and visual acuity.

		Precision			Recall			F-score		
		**Ref.**	**Non-ref.**	**Non-grad.**	**Ref.**	**Non-ref.**	**Non-grad.**	**Ref.**	**Non-ref.**	**Non-grad.**
Age	<=40	33.33 (4.33-77.72)	97.95 (96.01-99.11)	18.18 (8.19-32.71)	20.00 (2.52-55.61)	91.41 (88.3-93.91)	66.67 (34.89-90.08)	25.00 (7.27-52.38)	94.57 (92.78-96.03)	28.57 (17.3-42.21)
	41-60	37.42 (34.06-40.88)	94.94 (94.48-95.36)	48.99 (46.41-51.57)	64.58 (60.03-68.94)	89.21 (88.60-89.79)	61.36 (58.51-64.15)	47.39 (44.60-50.18)	91.98 (91.60-92.35)	54.48 (52.56-56.38)
	61-70	26.74 (23.02-30.72)	89.13 (88.04-90.15)	60.83 (58.04-63.57)	62.01 (55.38-68.32)	80.09 (78.79-81.35)	65.41 (62.57-68.17)	37.37 (33.92-40.92)	84.37 (83.51-85.20)	63.04 (61.06-64.99)
	>70	13.80 (10.09-18.26)	83.26 (81.26-85.12)	73.49 (70.47-76.36)	58.57 (46.17-70.23)	74.61 (72.45-76.68)	69.30 (66.26-72.23)	22.34 (18.18-26.96)	78.70 (77.23-80.11)	71.34 (69.21-73.39)
Vision	Normal	30.39 (27.69-33.19)	93.31 (92.85-93.74)	52.96 (50.98-54.92)	61.61 (57.39-65.7)	86.73 (86.14-87.3)	62.15 (60.06-64.21)	40.70 (38.32-43.11)	89.9 (89.52-90.26)	57.19 (55.75-58.61)
	VI	28.31 (24.17-32.75)	88.92 (87.61-90.13)	69.64 (66.49-72.65)	63.00 (55.91-69.7)	81.56 (80.04-83.01)	67.95 (64.81-70.98)	39.07 (35.28-42.96)	85.08 (84.08-86.04)	68.78 (66.58-70.92)
	Severe VI	50.00 (29.12-70.88)	86.90 (77.78-93.28)	74.68 (63.64-83.8)	100.00 (73.54-100)	71.57 (61.78-80.06)	80.82 (69.92-89.1)	66.67 (49.03-81.44)	78.49 (71.89-84.17)	77.63 (70.17-83.98)
	Blind	28.12 (13.75-46.75)	79.59 (70.26-87.07)	80.28 (72.78-86.48)	75.00 (42.81-94.51)	65.00 (55.76-73.48)	81.43 (73.98-87.5)	40.91 (26.34-56.75)	71.56 (65.08-77.45)	80.85 (75.77-85.27)
Total		29.6 (27.40-31.88)	92.56 (92.13-92.97)	58.58 (56.97-6019)	62.69 (59.17-6612)	85.65 (85.11-86.18)	65.06 (63.40-66.69)	40.22 (38.25-42.21)	88.97 (88.62-89.31)	61.65 (60.50-62.80)

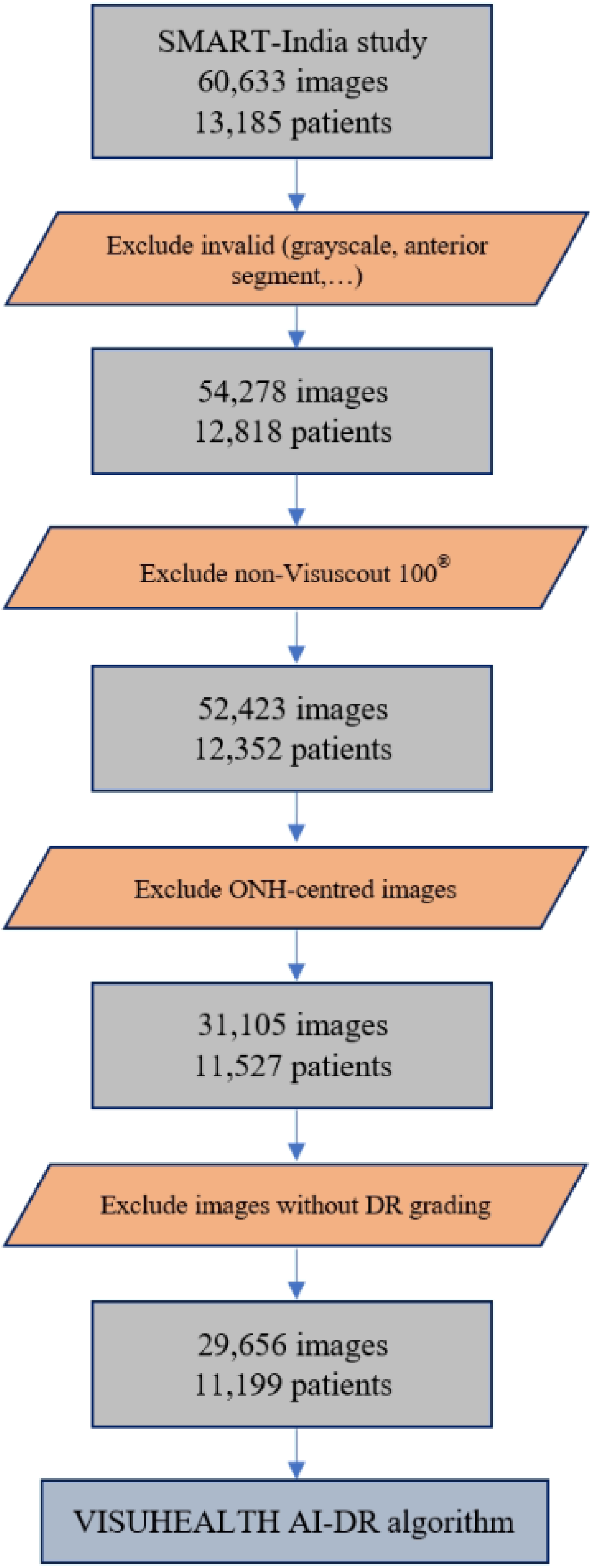

Figure S1. Study participants and data curation.
